# REV-ERB activation as a novel pharmacological approach for treating inflammatory pain

**DOI:** 10.3389/fphar.2023.1171931

**Published:** 2023-04-19

**Authors:** Sangeet Makhija, Joshua D. Griffett, Giri Babu Veerakanellore, Thomas P. Burris, Bahaa Elgendy, Kristine Griffett

**Affiliations:** ^1^ Department of Anatomy, Physiology, and Pharmacology, College of Veterinary Medicine, Auburn University, Auburn, AL, United States; ^2^ Center for Clinical Pharmacology, Washington University School of Medicine, University of Health Sciences & Pharmacy, St. Louis, MO, United States; ^3^ Department of Pharmaceutical and Administrative Sciences, University of Health Sciences & Pharmacy, St. Louis, MO, United States; ^4^ University of Florida Genetics Institute, Gainesville, FL, United States

**Keywords:** REV-ERB, inflammation, pain, NLRP3, SR9009, STL1267, inflammasome

## Abstract

Pain is a complex problem affecting millions of people worldwide. The current therapies to reduce pain are limited as many treatment options inadequately address the causes of pain, lead to tolerance of the drug, or have adverse effects including abuse potential. While there are many causes of pain, one underlying mechanism to the pathogenesis and maintenance of pain conditions is chronic inflammation driven by the NLRP3 inflammasome. Several inflammasome inhibitors are currently under investigation however have the potential to suppress the functioning of the innate immune system, which may cause unwanted affects in patients. Here, we show that the nuclear receptor REV-ERB can suppress the activation of the inflammasome when pharmacologically activated with small molecule agonists. Additionally, REV-ERB activation appears to have analgesic potential in a model of acute inflammatory pain, likely as a result of inflammasome suppression.

## 1 Introduction

Chronic pain affects the lives of people worldwide and has significant implications on healthcare (Eccles and Davies, 2021). Conventional therapeutics oftentimes are insufficient in long-term pain management and can result in an array of adverse effects (Janakiram et al., 2019). As pain accompanies a wide variety of chronic diseases and is highly subjective, it is important to target pathways that play a significant role in the development and maintenance of pain stemming from different conditions. The NLR family pyrin domain containing 3 (NLRP3) inflammasome has been implicated as a major driver of pain by increasing the processing of the pro-inflammatory cytokine, interleukin 1 beta (IL-1β) (Elzinga et al., 2019). Chronic activation of the inflammasome leads to upregulation of nuclear factor κB (NFκB)-driven inflammation which results in the recruitment of additional inflammatory molecules and cells (e.g., mast cells, neutrophils, microglia, etc.) and the initiation of an inflammatory microenvironment. Numerous studies have shown that regardless of the pain pathology, chronic activation of NLRP3 is always a factor in the development and maintenance of pain (Ren and Torres, 2009; Zhou and Zhou, 2014; Muley et al., 2016; Sommer et al., 2018; De Prá et al., 2019; Elzinga et al., 2019).

**FIGURE 1:**
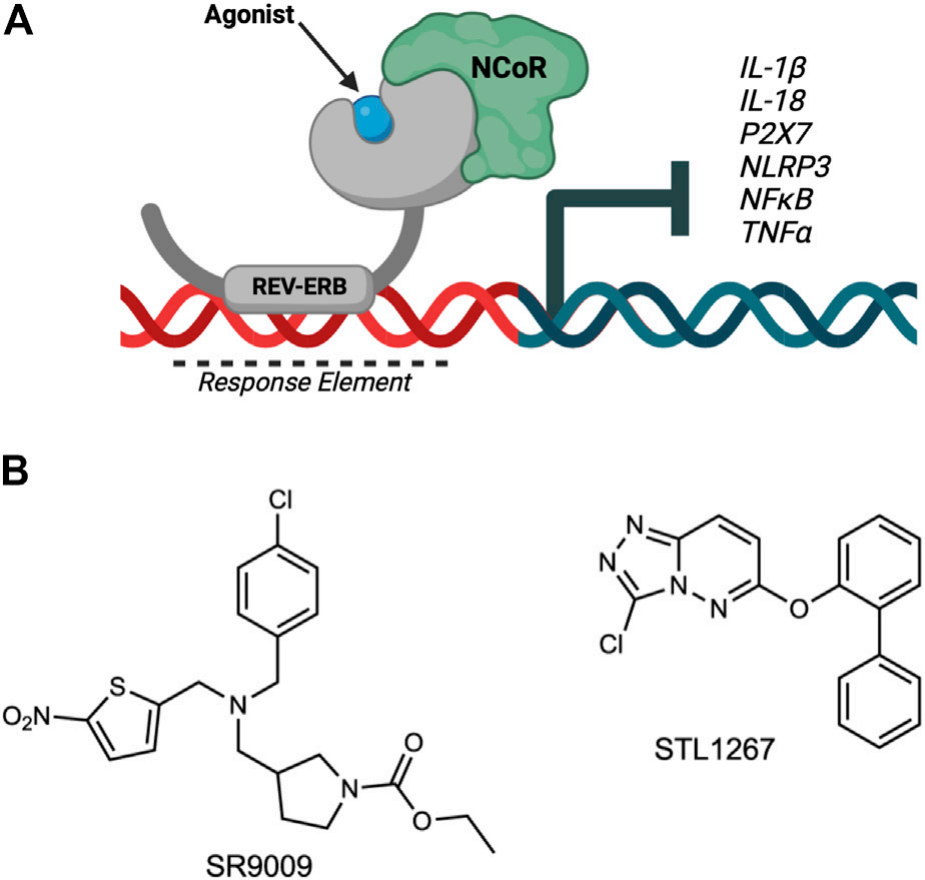
Overview of REV-ERB regulation of inflammatory components involved in pain. **(A)** The nuclear receptor REV-ERB recognizes sequences within the promoter of its target genes (Response Element) and binds DNA via its DNA-binding domain. Upon binding of a ligand, REV-ERB undergoes a conformational change and recruits the NCoR corepressor, causing the repression of transcription of its target genes including *NLRP3, IL-1β,* and other pro-inflammatory cytokines and pathway genes. **(B)** Structures of two REV-ERB agonists: SR9009 and STL1267.

REV-ERBα and REV-ERBβ (encoded by the genes *NR1D1 and NR1D2,* respectively) are members of the nuclear receptor superfamily of transcription factors (Raghuram et al., 2007). The REV-ERBs regulate a wide variety of physiological processes including circadian rhythm, lipid and glucose metabolism, and inflammatory pathways of the innate and adaptive immune systems. Both REV-ERBs are quite unique from the other nuclear receptors as they lack a carboxy-terminal activation of function-2 region from their ligand binding domains, and therefore can only act as a repressor of transcription by recruiting the nuclear receptor co-repressor (NCoR) (Figure 1A). Because of this, REV-ERB agonists enhance repression of direct target genes.

REV-ERB activity can be modulated with small molecule ligands to drive changes in the transcription of its direct target genes. For example, several studies demonstrated that the REV-ERB agonist SR9009 can effectively suppress the transcription of *IL-1β* and *NLRP3,* two genes that are REV-ERB targets (Pourcet et al., 2018; Duez and Pourcet, 2021). Based on this role of REV-ERB in regulating inflammatory pathways, we sought to evaluate if suppression of the inflammasome via REV-ERB activation had analgesic potential.

Recently, we described a novel small molecule agonist for REV-ERB, STL1267, with greater potency and selectivity for REV-ERB than SR9009 (Murray et al., 2022). For our studies, we compared the STL1267 compound to the first-generation *in vivo* REV-ERB agonist, SR9009, to evaluate direct REV-ERB effects on inflammation and pain. In the current study, we first validated the previous work demonstrating that REV-ERB regulates inflammatory processes by confirming effects in bone marrow-derived macrophages in *Nr1d1* ^*−/−*^ mice, as well as in human monocytes. We then investigated whether pharmacological activation of REV-ERB by small molecule ligands (SR9009 and STL1267) could suppress activation of the inflammasome in human microglia cells, which are more relative to pain associated with nerve damage. Finally, we confirmed our findings in an acute inflammatory pain model to demonstrate that activation of REV-ERB has efficacy as an inflammatory pain therapeutic.

## 2 Methods

### 2.1 Compounds

Both SR9009 (Solt et al., 2012) and STL1267 (Murray et al., 2022) (Figure 1B) were synthesized in house as previously described.

### 2.2 Mice

*Nr1d1* knockout mice and wildtype counterparts were obtained from Jackson Labs (Bar Harbor, ME) and maintained in compliance with IACUC guidelines at Washington University in St. Louis and Auburn University College of Veterinary Medicine. Bone marrow-derived macrophages (BMDMs) were collected following rapid cervical dislocation from femurs and tibias and immediately analyzed by quantitative polymerase chain reaction (QPCR).

### 2.3 Cell culture and inflammatory marker expression analysis

The human monocyte cell line, Thp-1, was purchased from ATCC (Catalog #TIB-202), maintained in a humidified incubator at 37°C with 5% CO2, and cultured in antibiotic-free RPMI media supplemented with 10% FBS. Cells were tested for *mycoplasma* regularly as a quality control. To differentiate Thp-1 cells, cells were primed for 24 h in media supplemented with Paramethoxyamphetamine (PMA; Sigma P1585). After priming, cells were washed and plated in fresh PMA-free culture media in 100 mm polystyrene dishes for 72 h. For the last 24-h, dimethylsulfoxide (DMSO), SR9009 (10 μM), or STL1267 (10 μM) was added to the media as a “pre-treatment.” The media was replaced with culture media containing 20 ng/mL interferon gamma (IFNγ) and 250 ng/mL lipopolysaccharide (LPS) for 48 h.

To determine whether SR9009 or STL1267 could suppress the release of IL-1β, we performed an ELISA (ThermoFisher A35574) as per manufacturer’s protocol (*n* = 6) from media removed from the cell culture dishes. For gene expression analysis, total RNA was isolated from the cells using Qiagen RNeasy kit per manufacturer’s protocol. Bio-Rad iScript cDNA synthesis kit was used to reverse transcribe up to 1 μg of cDNA for QPCR analysis using SYBR green (Invitrogen Power SYBR) using the ddCt method on the QuantStudio5 384-well QPCR system (Applied Biosystems). Genes normalized to 36b4 housekeeping gene for all experiments using fold-change analysis and plotted on Graphpad Prism software.

The human microglia cell line, HMC3, were a gift from Dr. Rajesh Amin. Cells were cultured in DMEM with 10% FBS (Corning) and 2.5 mM L-Glutamine (Gibco) without antibiotics and maintained in a humidified chamber at 37°C with 5% CO_2_. Cells were tested for *mycoplasma* regularly as a quality control. Cells were seeded in 12-well tissue culture-treated plates (Corning) at 1,00,000 cells/mL in normal cell media. The following day, cells were pre-treated with either DMSO, SR9009, or STL1267 at 10 μM and incubated overnight. The next day, cells were stimulated with 100 ng/mL LPS or Media (control) in the continued presence of compound treatment. Media was collected the following day for ELISA analysis and cells were either immunostained or collected for QPCR as described above

### 2.4 QPCR primers

Primers were purchased from Integrated DNA Technologies (IDT) and checked for specificity using NCBI Primer-BLAST as follows:

Human *NLRP3* Forward: 5′-CAT​GAG​TGC​TGC​TTC​GAC​AT-3′
Human *NLRP3* Reverse: 5′-CGA​CTC​CTG​AGT​CTC​CCA​AG-3′
Human *36B4* Forward: 5′- CGA​CCT​GGA​AGT​CCA​ACT​AC-3′
Human *36B4* Reverse: 5′- ATC​TGC​TGC​ATC​TGC​TTG-3′
Human *Il-1β* Forward: 5′- CAC​CAC​TAC​AGC​AAG​GGC​TTC​A-3′
Human *Il-1β* Reverse: 5′- GCA​TCT​TCC​TCA​GCT​TGT​CCA​T-3′
Human *Il-18* Forward: 5′- TCC​TTG​ATG​TTA​TCA​GGA​GGA​TTC​A-3′
Human *Il-18* Reverse: 5′- GCT​GTA​ACT​ATC​TCT​GTG​AAG​TGT​G-3′
Mouse *Il-1β* Forward: 5′-GCA​ACT​GTT​CCT​GAA​CTC​A-3′
Mouse *Il-1β* Reverse: 5′-CTC​GGA​GCC​TGT​AGT​GCA​G-3′
Mouse *Il-18* Forward: 5′- CTG​GAG​CTG​CTG​ACA​GGC​CTG​A-3′
Mouse *Il-18* Reverse: 5′- GCC​CAG​GAA​CAA​TGG​CTG​CCA​T-3′
Mouse *Nlrp3* Forward: 5′- GCC​TCA​CCT​CAC​ACA​GCT​GCT​G-3′
Mouse *Nlrp3* Reverse: 5′- TTG​GCG​ATC​TGT​GCG​TGG​TGA​C-3′
Mouse *36b4* Forward: 5′- AGA​TTC​GGG​ATA​TGC​TGT​TGG​C-3′
Mouse *36b4* Reverse: 5′- TCG​GGT​CCT​AGA​CCA​GTG​TTC-3′

### 2.5 Immunostaining HMC3 cells for NLRP3

Following removal of the cell media, cells were rinsed three times with cold 1X PBS, then fixed with 4% paraformaldehyde (PFA) in phosphate buffered saline (PBS; pH 7.4) for 10 min at 37°C. PFA was removed and the cells were gently washed three times with 1X PBS. Cells were permeabilized by incubating in 0.1% Triton X-100 in 1X PBS at room temperature for 15 min. Following permeabilization, cells were again washed in 1X PBS three times then blocked in 2% BSA in 1X PBS at room temperature for 1 h. The NLRP3 primary antibody (ThermoFisher MA5-32255) was diluted per manufacturer’s protocol in 0.1% bovine serum albumin (BSA) in 1X PBS and incubated for 3 h at room temperature. Cells were washed three times with 1X PBS then incubated with secondary antibody (per manufacturer’s protocol; ThermoFisher A11008) and counterstained with phalloidin (ThermoFisher B3475) in 0.1% BSA in 1X PBS for 45 min. Cells were washed three times with 1X PBS and fluoromount media (ThermoFisher P36971) was added to each well to cover the cells. The Discover Echo Revolve microscope system was used to image the cells.

### 2.6 *λ-*Carrageenan injections for acute inflammatory pain

All animal housing and research procedures involving rats were done at CARE Research, LLC. The standards for animal husbandry and care followed are those found in the Guide for the Care and Use of Laboratory Animals, eighth edition, Revised 201 (the Guide). Animal welfare for this study was in compliance with the U.S. Department of Agriculture’s (USDA) Animal Welfare Act (9 CFR Parts 1, 2, and 3), the Guide and CARE Research SOPs. Twenty-one female Sprague-Dawley rats were purchased from Charles River and allowed to acclimate to the testing facility for 7 days. Prior to dosing, all rats were subjected to baseline withdrawal response behavior testing (Von Frey filament and thermal response threshold to a heat source) and footpad thickness measurements using a digital caliper on the left hindfoot. Body weights were recorded pre-dose to determine appropriate dosing volumes. On the first day of the experiment, rats were administered a single dose of either vehicle control (10% DMSO; 12% CremophoreEL; 78% PBS), 100 mg/kg SR9009, or 50 mg/kg STL1267 via intraperitoneal injection. Dosing was determined by our previous work in which we evaluated different vehicles and performed pharmacokinetic analysis (Murray et al., 2022). Injection of compounds occurred as close to “lights on” as possible (i.e., 7 a.m.). One hour after compound administration, rats were given an injection of *λ*-Carrageenan into the left hind paw (1cc syringe loaded with 100ul of 2.0% carrageenan (w/v), subcutaneous injection into plantar footpad) under light isoflurane anesthesia to induce the inflammatory response. Since *λ*-Carrageenan is reported to induce peak inflammatory response within 3–5 h, the first set of testing occurred within this period. Testing then occurred again at approximately 24 h post-injection (hpi) of λ-carrageenan and finally at approximately 48-hpi.

### 2.7 Von Frey filament testing

Withdrawal responses were measured using Von Frey filaments at the injection site at baseline testing, ∼4-hpi, ∼24-hpi, and ∼48-hpi for all 21 rats. The “ascending stimulus” method was used to determine an estimate for the mechanical threshold. For testing, the rats were placed in clear metabolism cages with the base removed to give access to the wire mesh floor. While in the testing chamber, Von Frey filaments were applied to the plantar surface of the injected hind paw in a series of ascending forces (von Frey filaments are plastic hairs of calibrated diameters, 5 cm long and are fixed on hand-held applicators). The chosen filament was applied to the plantar surface of the hind left paw until the filament was seen to bend. The procedure was repeated five consecutive times on the left hind foot of each animal. The expected response was a paw withdrawal, sudden flinching, or paw licking. The response was considered positive if at least three expected responses were observed out of five trials. The procedure was started again on each animal with the next filament of greater force. The procedure was repeated until a rat gave a positive response on two consecutive filaments. Once a positive response was given on two consecutive filaments, testing was stopped on that animal. The gram value of the lower filament that gave a positive response was considered the paw withdrawal threshold for the animal.

### 2.8 Thermal response threshold to a heat source (Hot plate)

In this test, the surface of the heat source was heated to a constant temperature of approximately 55°C verified by an infrared thermometer. A large glass beaker (10,000 mL) was placed on the heat source and the bottom of the beaker measured for temperature with the infrared thermometer. Each rat was placed into the beaker and a timer was activated. Each rat remained on the heat source for 30 s. During that time, the number of times the carrageenan injected hind left foot was lifted from the heat source was recorded. If there was no response observed by 30 s, the test was stopped, and the rat returned to its cage.

### 2.9 Footpad thickness measurements

Footpad thickness was measured from the top of the foot to the base of the central pad by digital calipers.

### 2.10 Statistics

All data was analyzed using Graphpad Prism 9 as indicated in the figure legends. Significance is indicated by: *(*p* ≤ 0.05), **(*p* ≤ 0.01), ***(*p* ≤ 0.001), and ****(*p* ≤ 0.0001).

## 3 Results

### 3.1 REV-ERB agonists suppress pro-inflammatory cytokines and the NLRP3 inflammasome in LPS-stimulated macrophages

**FIGURE 2:**
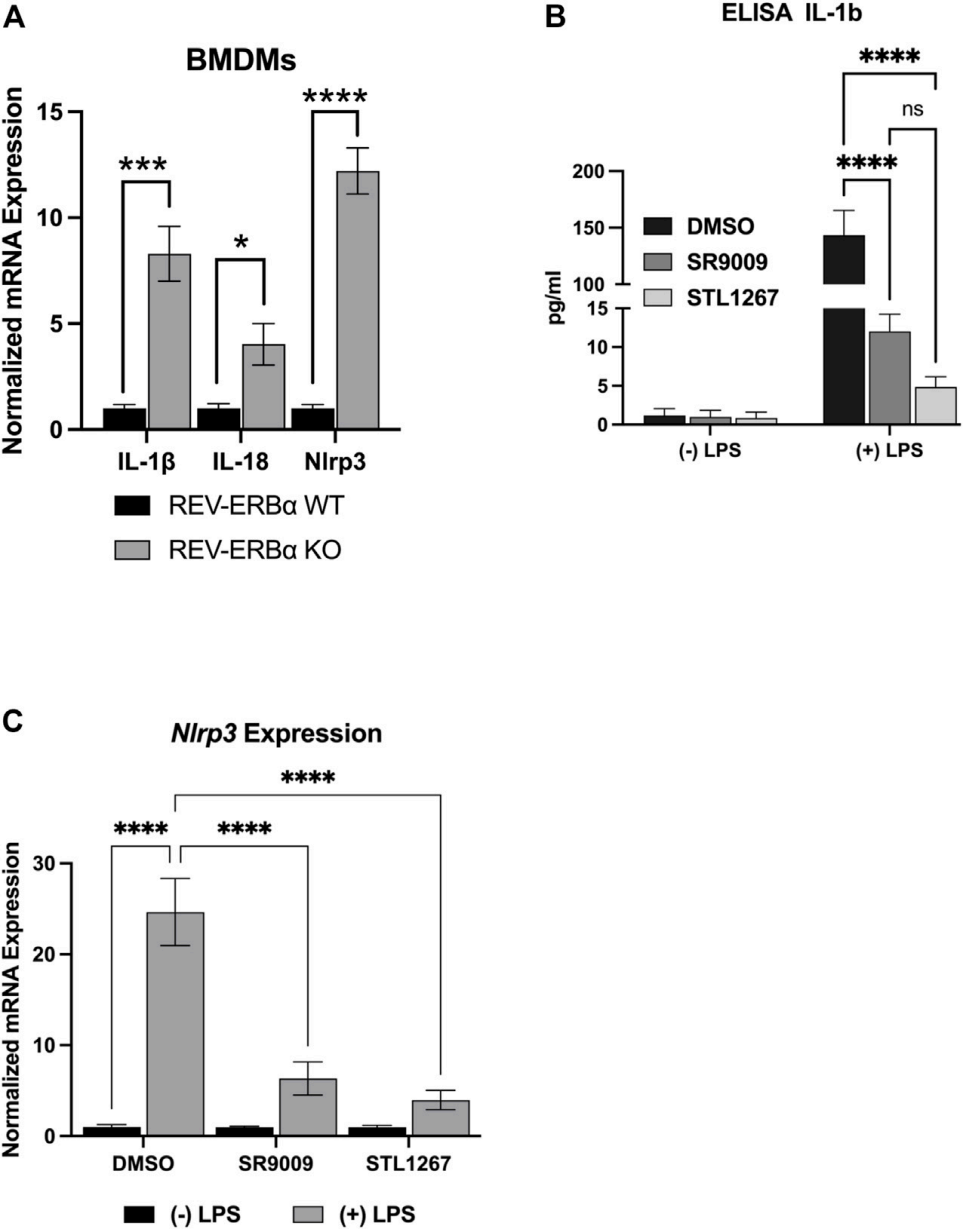
*In vitro* model of REV-ERB anti-inflammatory actions. **(A)** Mouse bone marrow-derived macrophages from *Nr1d1* ^*−/−*^ (*n* = 7) and wildtype (*n* = 12) mice. Both male and female mice were used for this study and no sex-specific differences were noted. **(B)** IL-1β ELISA results for differentiated Thp-1 cells. **(C)** *NLRP3*gene expression results of the differentiated Thp-1 cells. Results were graphed and analyzed in Graphpad Prism by ANOVA and represented as Mean ± SEM.

Previous studies have shown that activating REV-ERB, either pharmacologically or by overexpression, can suppress IL-1β and other pro-inflammatory cytokines in several inflammatory models (Gibbs et al., 2012; Pourcet et al., 2018; Guo et al., 2019; Morioka et al., 2019; Hong et al., 2021). Conversely, genetic loss of function of REV-ERB leads to enhanced IL-1β expression. Here, we wanted to evaluate the efficacy of the novel REV-ERB agonist STL1267 to suppress the NLRP3 inflammasome in LPS-stimulated macrophages as compared to SR9009. First, we validated previous studies that demonstrated the loss of REV-ERBα led to an increase in the expression of pro-inflammatory molecules including NLRP3. We assessed the pro-inflammatory markers, *IL-1β*, *IL-18*, and *Nlrp3* in bone marrow-derived macrophages (BMDMs) from *Nr1d1* ^*−/−*^ mice and wild-type mice by QPCR. As shown in Figure 2A, mice lacking REV-ERBα had significant increases in the expression of the three pro-inflammatory genes, suggesting that the activation of REV-ERB suppresses inflammation.

We then investigated the action of SR9009 and STL1267 on suppression of the inflammasome by differentiating Thp-1 monocytes by priming with PMA, allowing the cells to recover in PMA-free media, then stimulating the cells with LPS and IFNγ. During the last 24-h of the recovery period, either DMSO, 10 μM SR9009, or 10 μM STL1267 was added to the media as a “pre-treatment.” Control cells were pre-treated with compounds but not stimulated with LPS. We then evaluated IL-1β release by removing media and performing an ELISA. As Figure 2B shows, Thp-1 cells that did not receive LPS stimulation secreted low amounts of IL-1β regardless of the pre-treatment. As expected, we observed a significant increase in IL-1β in DMSO-treated LPS-stimulated cells. Moreover, the REV-ERB agonists SR9009 and STL1267 both significantly reduced the amount of secreted IL-1β in the LPS-stimulated cells, validating that REV-ERB activation is anti-inflammatory. Total RNA was isolated from the same cells to evaluate changes in gene expression of various pro-inflammatory cytokines and the NLRP3 inflammasome. Figure 2C shows that upon LPS stimulation of the cells, the expression of *Nlrp3* is significantly increased however both REV-ERB agonists significantly suppress the expression of the inflammasome in this model.

### 3.2 NLRP3 inflammasome activation is suppressed in human microglia by REV-ERB agonists

Once we validated previous studies that demonstrated REV-ERB regulates inflammasome activation and expression of several pro-inflammatory molecules, we then evaluated the anti-inflammatory activity in a human microglia cell line, HMC3. As resident macrophages of the central nervous system (CNS), microglia have been implicated in the activation and sensation of pain in humans (Clark et al., 2010; Rhie et al., 2020; Ren and Illes, 2022). We hypothesized that the suppression of these factors via REV-ERB pharmacological activation may provide analgesic effects by reducing inflammation. To test our hypothesis that REV-ERB agonists suppress inflammation in microglia, we performed a similar experiment as described with the Thp-1 cells.

**FIGURE 3:**
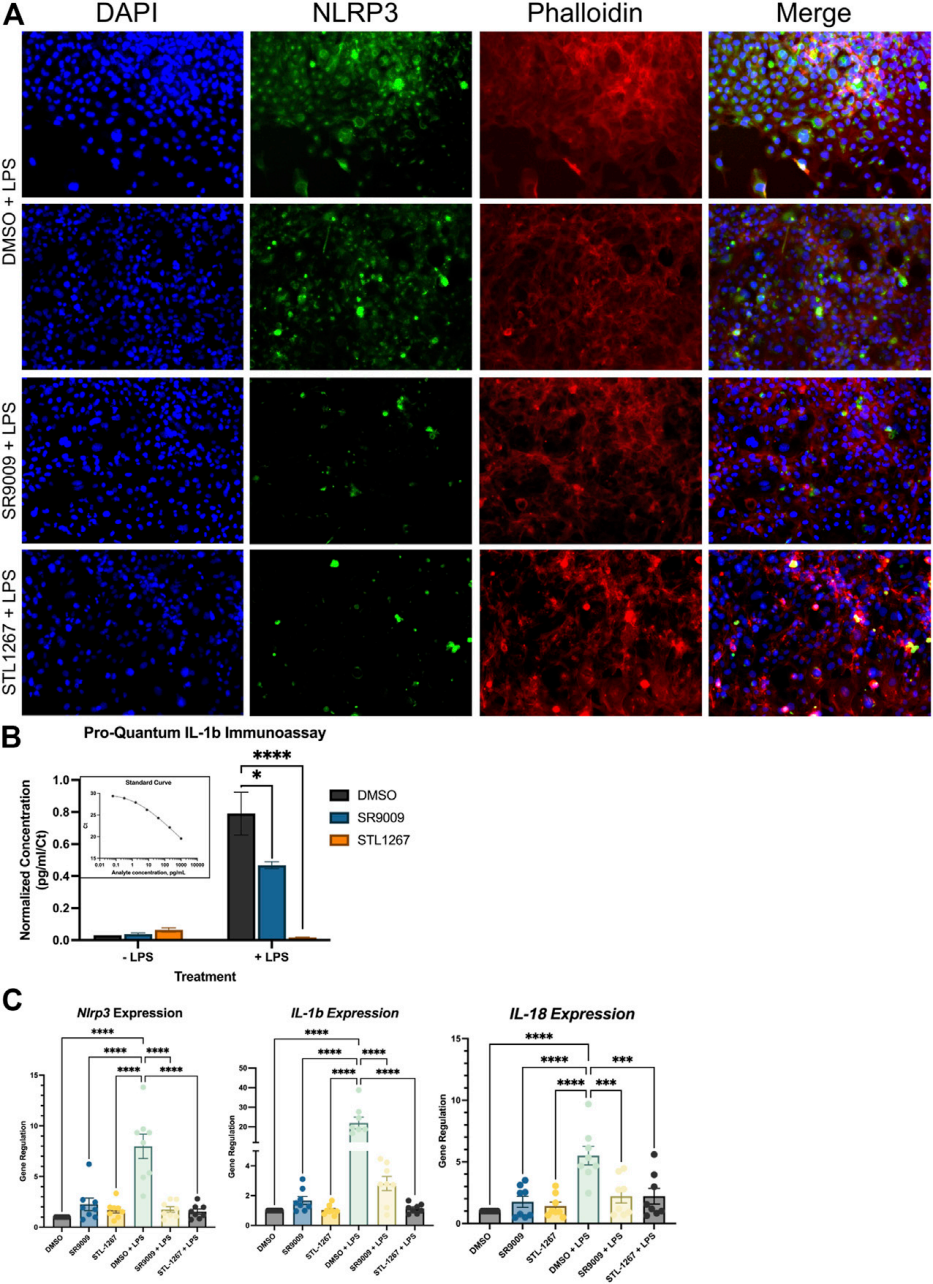
REV-ERB agonists have an anti-inflammatory effect on a human microglia cell line*.* **(A)** Immunostaining of LPS-stimulated HMC3 cells treated with either DMSO, SR9009, or STL1267. Cells were immunostained for NLRP3 (green) and counterstained with DAPI (Blue) and Actin (Phalloidin; Red) to verify cellular occupancy of NLRP3. **(B)** ELISA assay evaluating the secretion of IL-1β into the cell culture media. Results are shown as pg/mL normalized to C_t_ value of IL-1β per the ThermoFisher protocol using the Connect cloud software and graphed in Graphpad Prism. **(C)** Gene expression results for *NLRP3, IL-1β*, and *IL-18*. For all cell-based experiments, an *n* = 6 for each condition was used. Experiments were performed on three different occasions by different researchers, and the results were analyzed separately by ANOVA and represented by Mean ± SEM.

HMC3 cells were pre-treated with either DMSO, 10 μM SR9009, or 10 μM STL1267 for 24-h, then stimulated with LPS for an additional 24-h. Media was collected for ELISA analysis while the cells were fixed and immunostained for NLRP3 or collected for QPCR analysis. As shown in Figure 3A, we observed a significant decrease in NLRP3 staining in both the SR9009 and STL1267-treated cells as compard to DMSO treated cells, which is consistent with our previous Thp-1 cell data. We also observed no significant changes in cells without LPS-stimulation (Supplementary Figure S1). A control containing no primary antibody was also performed to confirm the green signal was due to the presence of NLRP3 (Supplementary Figure S2). The media was collected from all the plates and we performed the Pro-Quantum IL-1β immunoassay to evaluate whether the REV-ERB agonists could suppress the secretion of IL-1β, a key mediator of inflammation and NLRP3 activation. We found that while 10 μM SR9009 significantly suppressed the secretion of this cytokine following LPS stimulation, and STL1267 pre-treatment reduced IL-1β to levels consistent with no LPS stimulation (Figure 3B). This clearly shows that the potency and efficacy of the STL1267 is superior to that of SR9009 in this model, consistent with our previous pharmacological characterization of STL1267 (Murray et al., 2022). We then performed QPCR to evaluate the expression of *NLRP3, IL-1β,* and *IL-18* in the cells following REV-ERB agonist treatment. As shown in Figure 3C, both REV-ERB agonists significantly suppress the expression of *NLRP3, IL-1β,* and *IL-18* in the LPS-stimulated cells. Our data suggests that not only is REV-ERB a regulator of inflammation in the periphery as described by numerous labs, the pharmacological activation of REV-ERB has significant effects on suppressing inflammation in the CNS, which may play a role in pain and other chronic inflammatory conditions (Gibbs et al., 2012; Amir et al., 2018; Pourcet et al., 2018; Baxter and Ray, 2019; Chang et al., 2019; Amir et al., 2020; Hong et al., 2021; Gray and Gibbs, 2022).

### 3.3 A single dose of REV-ERB agonist reduces pain behavior in Sprague-Dawley rats

**FIGURE 4:**
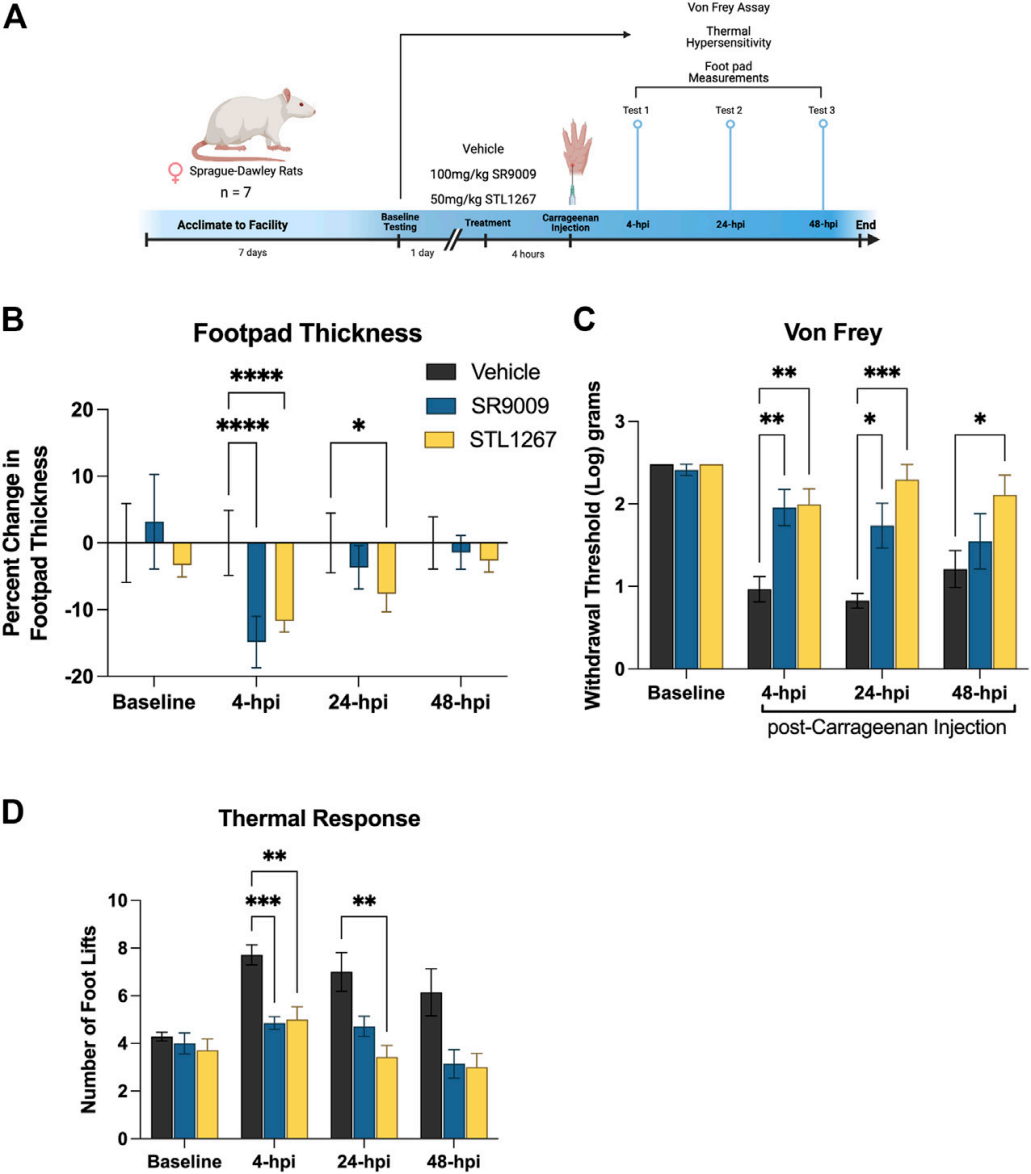
REV-ERB agonists suppress pain behavior in a rat model of acute inflammatory pain. **(A)** Schematic of the rat model used to evaluate the efficacy of REV-ERB agonists SR9009 and STL1267 in acute inflammatory pain. **(B)** Measurement of the inflammatory response following a single injection of REV-ERB agonist (i.p.) and carrageenan injection in the rat hind paw. Measurements were taken with a digital caliper and normalized as the change in thickness as a percentage to the baseline measurements. **(C)** Mechanical hypersensitivity was measured using a Von Frey filament. **(D)** Thermal hypersensitivity was also tested on these animals following a single injection of REV-ERB agonist (i.p.) and evaluated by recording the number of foot lifts over a 30-s interval. Data was analyzed in Graphpad Prism using ANOVA with Dunnett’s post-hoc test and represented by Mean ± SD.

Given that inflammation plays a major role in the development of pain conditions, we sought to investigate whether suppressing the NLRP3 inflammasome via the REV-ERB agonists SR9009 and STL1267 will be analgesic in an inflammatory pain model. To perform these assays, we used 21 female Sprague-Dawley rats and assessed baseline sensitivity to both mechanical and thermal stimuli by Von Frey filament assay and hot plate. The following day, rats were administered either vehicle, SR9009, or STL1267 via intraperitoneal injection, rested for 1 h, then administered *λ*-Carrageenan subcutaneously into the left footpad to induce the acute inflammatory response (saline was injected into the right footpad as an internal control) (Allen and Yaksh, 2004; McCarson, 2015). This method produces a localized inflammatory response characterized by redness, swelling, het, reduced function, and hypersensitivity (as demonstrated by hyperagesia and/or allodynia), and can be used to assess efficacy of anti-inflammatory molecules (Fehrenbacher et al., 2012). At 4-, 24-, and 48-hpi, rats were assessed for mechanical and thermal hypersensitivity, and footpad thickness as shown in Figure 4A.

Measurments of footpad thickness show that a single administration of REV-ERB agonist (either SR9009 or STL1267) was sufficient to reduce footpad inflammation driven by *λ*-Carrageenan injection at 4-hpi (Figure 4B). STL1267 continued to suppress footpad inflammation at 24-hpi, suggesting that it may be more efficacious at the 50 mg/kg dose as compared to SR9009 which was given at 100 mg/kg, as determined by our previous work with these compounds (Solt et al., 2012; Murray et al., 2022). By 48-hpi, there was no significant change in footpad thickness as compared to the vehicle control group, which was expected given the rats only received a single dose of REV-ERB agonists and the compounds should have been completely metabolized by this time.

Mechanical hypersensitivity evaluated by Von Frey filaments is a standard assay performed to evaluate analgesic activity of compounds (Allen and Yaksh, 2004).The withdrawal threshold measurement at baseline suggests that there were no differences among groups, however at 4- and 24-hpi both groups treated with REV-ERB agonists displayed significantly higher withdrawal thresholds as compared to the vehicle control group (Figure 4C). At 48-hpi, although footpad thickness was not significantly affected, the group administered the single dose of STL1267 displayed a significant increase in paw withdrawal threshold as compared to the vehicle control group, suggesting that the administration of REV-ERB agonists may provide analgesic activity in inflammatory pain models.

Evaluating the reponse to a thermal stimulus represents an additional aspect of quantitative sensory testing, which may facilitate the findings of our REV-ERB compounds in a translational manner. Thermal hyperalgesia is a common pathology in a number of human pain states, and is often used to evaluate novel small molecules. We evaluated the thermal response threshold of the animals using a hotplate set to 55°C. The number of foot lifts over a thirty-second period was recorded and shows that administration of REV-ERB agonists significantly reduced the number of foot lifts at 4-hpi (Figure 4D). At 24-hpi, SR9009 reduced the number of foot lifts but was not significant compared to the vehicle group. However, STL1267 did continue to show efficacy in this assay at 24-hpi. By 48-hpi, the number of foot lifts was still less than the control group although not significant.

## 4 Discussion

It is well known that chronic pain conditions are associated with inflammation and represents a major healthcare concern worldwide (Chen et al., 2021). Recent evidence points to chronic inflammation due to inflammasome activation in local inflamed tissues, peripheral nerves, or even the CNS (spinal cord) as a driver of the initiation and maintenance of chronic pain (He et al., 2019; Chen et al., 2021; Griffett, 2022). One such pro-inflammatory driver of pain is the cytokine IL-1β, which can incidentally contribute to pain by both increasing local inflammatory responses and by direct activation of nociceptors to elicit action potentials which initiates pain sensations (Chen et al., 2021). The activity of IL-1β is tightly regulated via the NLRP3 inflammasome. The inflammasome is a large multicomponent molecule that consists of NLRP3, ASC adaptors, and caspase enzymes, and is present primarily in immune cells including macrophages and microglia, although several recent studies have demonstrated its presence in neurons of the sensory nervous system (Baldwin et al., 2016; Caseley et al., 2020). The activation of the NLRP3 inflammasome is mediated through toll-like receptors (TLRs) and various cytokine receptors resulting in the NFκB-mediated upregulation of NLRP3 and IL-1β transcription. The chronic activation of NLRP3 has been implicated in a variety of disorders including atherosclerosis, auto-immune disorders (e.g., multiple sclerosis), and Alzheimer’s disease (Eren and Özören, 2019).

Previous studies have shown a direct regulation of both NLRP3 and IL-1β by REV-ERB. In collaboration with the Duez and Stalls group, we determined that there are several REV-ERB response elements in the promoter of the *Nlrp3* gene in mice, with corresponds with the human genome (Pourcet et al., 2018). The NLRP3 inflammasome is tightly regulated by a variety of physiological factors including circadian rhythms, as a direct result of REV-ERB (Duez and Pourcet, 2021). This work not only validated the direct control of inflammation in macrophages by REV-ERB, but also addressed the concepts that inflammation is a highly regulated process. Dysregulation of inflammatory mediators including NLRP3 can lead to a variety of diseases. Understanding the role that chronic NLRP3 inflammasome plays in the initiation and maintenance of pain conditions, we sought to evaluate whether the pharmacological activation of REV-ERB with small molecule agonists alleviated pain behaviors.

We first validated previously published data to confirm that loss of REV-ERB leads to increases in inflammatory mediators by evaluating the differences in pro-inflammatory cytokines (*IL-1β* and *IL-18*) and *Nlrp3* gene expression in mouse BMDMs from both REV-ERBα-null animals and wildtype (C57Bl/6J) animals. We confirmed that the loss of REV-ERB in these animals correlated with an increase in inflammatory molecule expression by QPCR. These results allowed us to develop our hypothesis that REV-ERB may play a role in pain driven by inflammation. We then wanted to evaluate the effects of pharmacological activation of REV-ERB in human monocytes, since these cells can recapitulate NLRP3 activity as seen in a variety of inflammatory diseases. We therefore used a standard procedure of differentiating Thp-1 cells towards an inflammatory macrophage phenotype and evaluated the effects of our small molecule REV-ERB agonists on inflammatory mediators. Our results not only validated previously published results demonstrating the anti-inflammatory effects of REV-ERB agonist SR9009, it also demonstrated that the new compound, STL-1267, has greater efficacy in these models as compared to SR9009 (Morioka et al., 2016; Morioka et al., 2021; Murray et al., 2022).

Since we are interested in the potential analgesic properties of REV-ERB agonists, we then decided to evaluate the effects on inflammatory molecules in a human microglia cell line. Microglia activation has been implicated as a key driver of a variety of pain conditions including chemotherapy-induced neuropathy, generalized inflammatory pain, migraine, and diabetic peripheral neuropathy pain (Sommer et al., 2018; Morioka et al., 2021). Upon nerve injury, microglia are capable of surrounding cell bodies of neurons and dorsal root ganglia, and serve as a source of inflammatory mediators (e.g., IL-1β, IL-18, TNF-α, chemokines, etc.) thereby directing a neuroinflammatory response. Without access to human primary microglia, the HMC3 cell line provides a translational approach to test whether the compounds can suppress neuroinflammation. Immunostaining for NLRP3 in these cells displayed a significant reduction of NLRP3 expression, suggesting that pharmacological activation of REV-ERB with small molecules can suppress NLRP3 activity in microglia. This finding has implications in numerous diseases in which microglia play a key role. We also compared expression of NLRP3 to cells that were not stimulated with LPS to confirm the immunostaining methods (Supplementary Figure S2). While these results are clear, we also confirmed the reduction of NLRP3 expression by evaluating the secretion of IL-1β by ELISA as well as determined that the REV-ERB agonists downregulated the expression of *NLRP3*, *IL-1β,* and *IL-18* by QPCR from these cell. From our results, we confirmed previous data suggesting that REV-ERB directly regulates the expression of *NLRP3* and hypothesized that if REV-ERB agonists can suppress the activation of inflammatory microglia, then we may observe analgesic activity in an inflammatory pain model.

To test this hypothesis, we contracted a research organization to blindly perform an acute inflammatory assay on rats in which the rats would only receive a single intraperitoneal injection of either vehicle, SR9009, or STL1267 an hour prior to carrageenan footpad injection. The CRO routinely performed these assays in rats, and this provided an additional model to validate the effects of REV-ERB activation in inflammatory pain, allowing us to confirm the translational value of REV-ERB. STL1267 is more soluble and stable in the vehicle as compared to SR9009, has increased potency as characterized in radioligand binding assays and luciferase co-transfection assays, and has improved pharmacokinetic properties and half-life (Murray et al., 2022).

The mechanical Von Frey test is a widely used method to evaluate mechanical allodynia in laboratory animals and remains to be the gold standard in determining mechanical thresholds in pain models. Likewise, the hotplate method can be used to evaluate heat thresholds (hypersensitivity) in animal models. Since a constant heat stimulus is applied, several nocifensive behaviors were used to evaluate the analgesic potential of the REV-ERB compounds including licking, jumping, hindpaw withdrawal, and leaning. We found that in the acute inflammatory model, a single dose of REV-ERB agonist was sufficient to reduce the initial inflammation that occurred as a result of footpad *λ*-Carrageenan injection. Interestingly, we observed significant differences in the withdrawal threshold following the carrageenan injection in both treatment groups suggesting that the compounds had an analgesic effect in the mechanical Von Frey assay. A similar effect was found in the thermal response assay, confirming our hypothesis that REV-ERB mediated repression of inflammatory processes has analgesic activities.

Pain is a multifaceted and diverse problem that can be attributed to a variety of pathologies. Likewise, evaluating pain in animal models presents some difficulties as we are limited to evaluating behaviors to assess pain levels. However, these models have provided key data that suggests there is translational value in targeting the REV-ERB receptor for inflammatory pain. While considerably more work needs to be performed to evaluate the role that REV-ERB plays in analgesia, we believe that this may have potential therapeutic implications in pain driven by inflammatory processes. Here, we explored how pharmacological activation of REV-ERB effects inflammatory processes of pain, mainly via macrophage and microglial-type processes of activating the NLRP3 inflammasome. These compounds will need to be explored in the future to evaluate the efficacy of analgesia in pain models less dependent on inflammatory processes, and the mechanisms involved are yet to be understood.

Given the issues of opioid misuse worldwide, it is imperative that new, non-opioid, effective therapeutics for chronic pain are developed and evaluated. One area that will need to be evaluated in the future is whether REV-ERB compounds that have efficacy in pain models affect the addiction or reward pathways. While we are not able to perform these experiments currently, we have previously published data that demonstrates the SR9009 series of REV-ERB agonists are non-addictive in a conditioned place preference (CPP) assay (Banerjee et al., 2014). That data also confirmed an “anti-addictive” effect of combining a REV-ERB agonist with cocaine in the CPP assay, suggesting that targeting REV-ERB for analgesia may be a safer alternative than opioid therapy (Banerjee et al., 2014). Further work in this area will need to be performed. Additionally, it is important to note that animal models have successfully predicted the usefulness of several commonly used treatments for pain including ibuprofen and morphine. The human processing and interpretation of pain is different from the animals commonly used in the laboratory. Likewise, disparities in therapeutic efficacy between rodents and humans may be attributed to differences in pharmacokinetics/pharmacodynamics (PK/PD) between species (Muley et al., 2016). While our current understanding of the translational value and predictive capacity of these compounds is limited, this work provides important biological information depicting the role that the nuclear receptor REV-ERB plays in inflammatory pain. Overall, the data presented here suggests that pharmacological activation of REV-ERB with small molecule ligands may have analgesic activity in models of acute inflammatory pain.

## Data Availability

The raw data supporting the conclusion of this article will be made available by the authors, without undue reservation.

## Supplementary material

SUPPLEMENTARY FIGURE S1 HMC3 cell immunostaining for NLRP3. These cells were not stimulated with LPS and served as a control for NLRP3 activity.

SUPPLEMENTARY FIGURE S2 HMC3 cell with the no primary antibody control. The fluorescent-conjugated secondary was used to control for off-target fluorescence of the immunostaining technique. The cells that were stimulated with LPS are on the top half, while the cells that received media are on the bottom half, separated by a black line.
