# Supplementary figures and images for: REV-ERB activation as a novel pharmacological approach for treating inflammatory pain
Source: Front Pharmacol. 2023 Apr 19;14:1171931. doi: 10.3389/fphar.2023.1171931 (PMC10154555; doi:10.3389/fphar.2023.1171931)

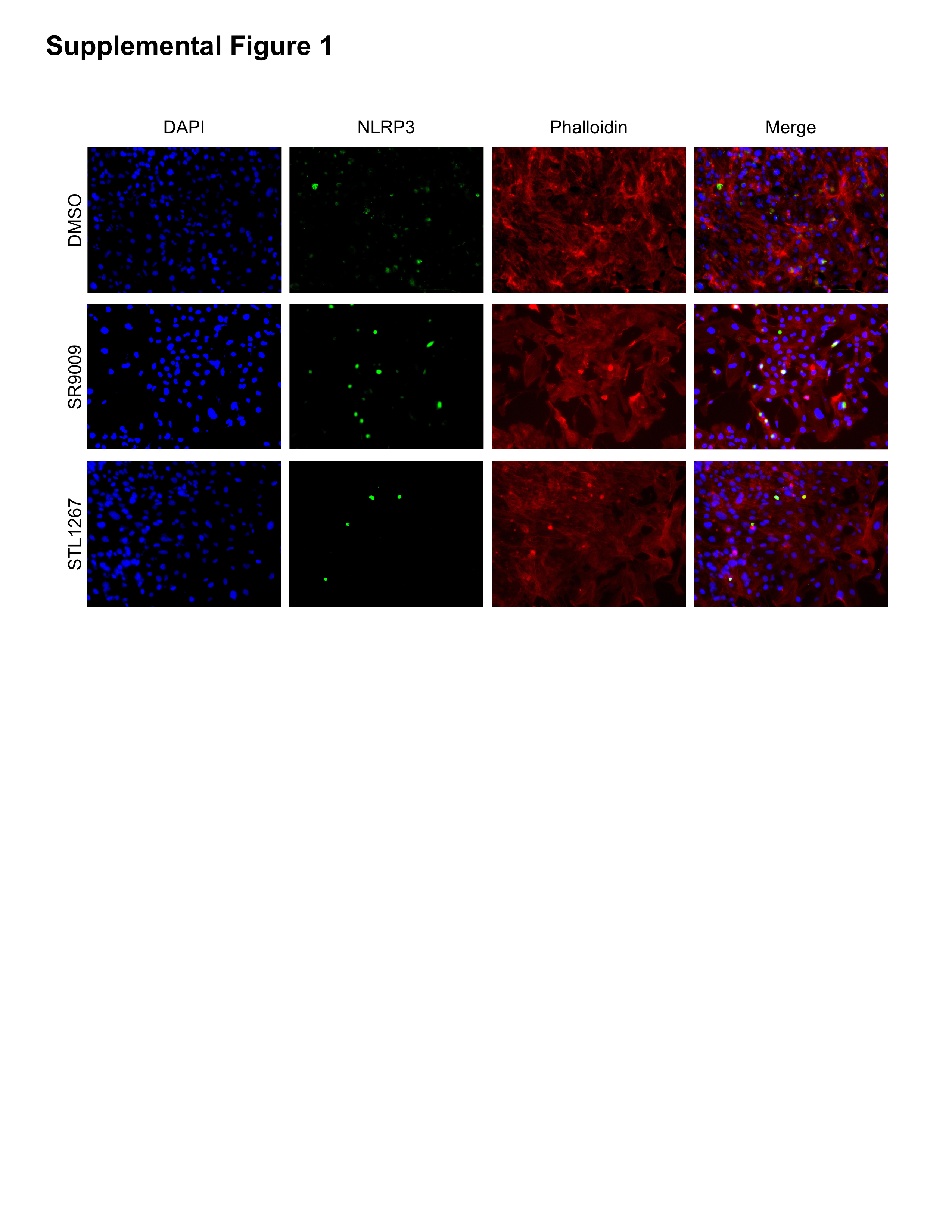

Supplement: Supplementary file 1 [file Image1.TIFF]

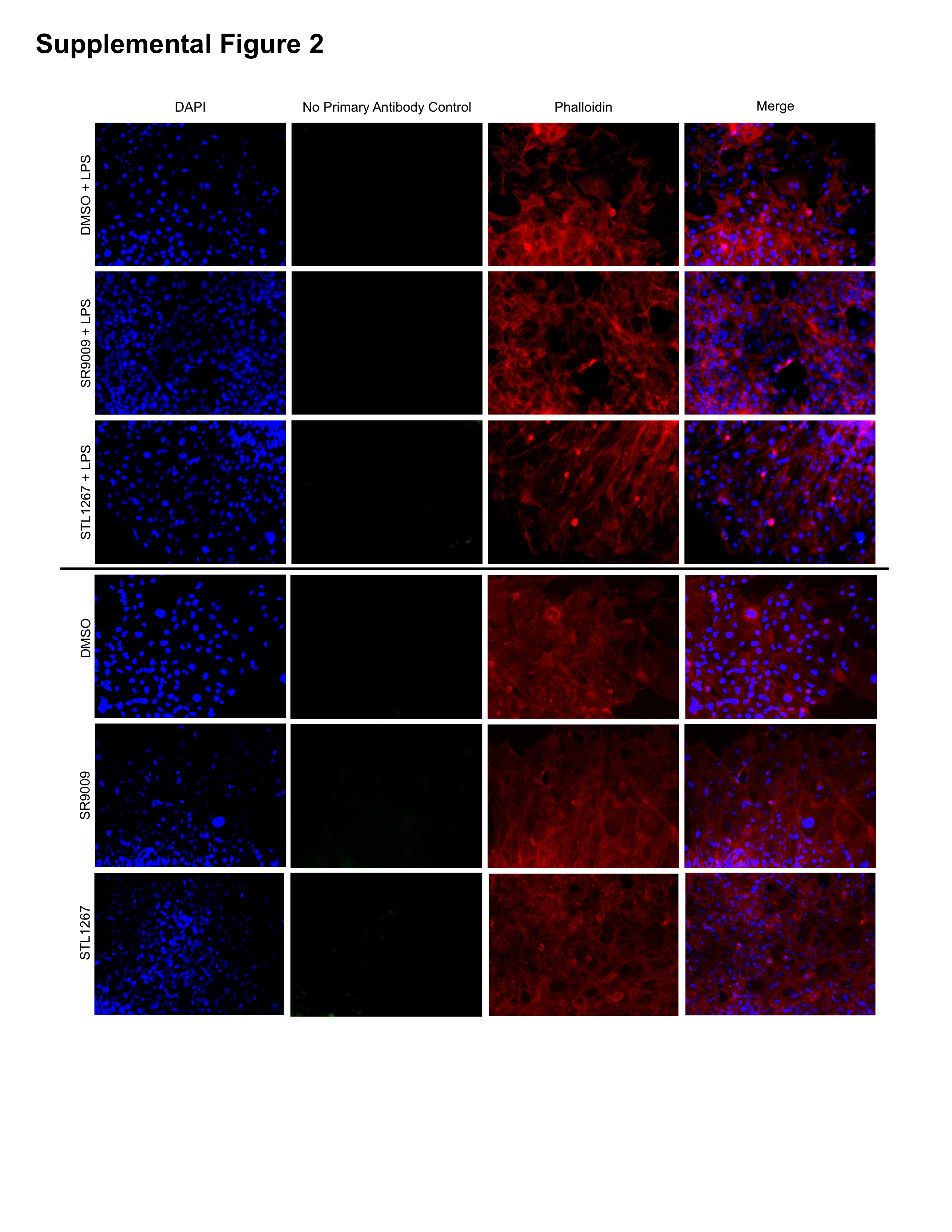

Supplement: Supplementary file 2 [file Image2.TIFF]
